# Assessing readiness factors for implementation of LGBTQ+ affirmative primary care initiatives: Practice implications from a mixed-method study

**DOI:** 10.3389/frhs.2022.901440

**Published:** 2022-08-24

**Authors:** Cathleen E. Willging, Marisa Sklar, Kristen Eckstrand, Robert Sturm, Sonnie Davies, Miria Kano

**Affiliations:** ^1^Pacific Institute for Research and Evaluation, Southwest Center, Albuquerque, NM, United States; ^2^Department of Psychiatry, University of California, San Diego, San Diego, CA, United States; ^3^Department of Psychiatry, University of Pittsburgh Medical Center, Pittsburgh, PA, United States; ^4^New Mexico Community AIDS Partnership, Santa Fe, NM, United States; ^5^Department of Internal Medicine, University of New Mexico Health Sciences Center, Albuquerque, NM, United States

**Keywords:** access, equity, gender minority, guidelines, health disparities, implementation, primary care, sexual minority

## Abstract

**Introduction:**

Access and utilization barriers in primary care clinics contribute to health disparities that disproportionately affect lesbian, gay, bisexual, transgender, and queer (LGBTQ+) people. Implementing inclusive practice guidelines in these settings may decrease disparities. The purpose of this exploratory/developmental study is to identify key issues affecting the readiness of primary care clinics to implement such guidelines.

**Methods:**

Using a concurrent mixed-method research design, we conducted surveys, interviews, and focus groups with 36 primary care personnel in clinics in New Mexico, USA, to examine readiness to implement LGBTQ+ inclusive guidelines, analyzing factors affecting motivation, general organizational capacity, and innovation-specific capacity. We supplemented these data by documenting LGBTQ+ inclusive policies and practices at each clinic. We undertook descriptive analyses and between-subscale comparisons controlling for within-rater agreement of the survey data and iterative coding and thematic analysis of the qualitative data.

**Results:**

Quantitatively, participants reported significantly more openness toward adopting guidelines and attitudinal awareness for developing LGBTQ+ clinical skills than clinical preparedness, basic knowledge, and resources to facilitate implementation. Six themes derived from the qualitative findings corroborate and expand on these results: (1) treating all patients the same; (2) addressing diversity in and across LGBTQ+ populations; (3) clinic climates; (4) patient access concerns; (5) insufficient implementation support; and (6) leadership considerations.

**Conclusion:**

This study demonstrates that personnel in primary care clinics support initiatives to enhance service environments, policies, and practices for LGBTQ+ patients. However, drawing on Iris Young's theory of structural injustice, we found that neutralizing discourses that construct all patients as the same and time/resource constraints may diminish motivation and capacity in busy, understaffed clinics serving a diverse clientele and reinforce inequities in primary care for LGBTQ+ people. Efforts are needed to build general and innovation-specific capacities for LGBTQ+ initiatives. Such efforts should leverage implementation teams, organizational assessments, education, leadership support, community engagement, and top-down incentives.

## Introduction

Decreasing health disparities for lesbian, gay, bisexual, transgender, and queer (LGBTQ+) people is a public health priority in the United States (U.S.) (1). Compared to their heterosexual and cisgender counterparts, LGBTQ+ people are more likely to suffer from poorer mental health, substance use, sexually transmitted infections, and other health conditions often identified in primary care. They are also less likely to access preventive services, cancer screening, and treatment for cardiovascular disease, diabetes, hypertension, and other serious conditions (1, 2).

LGBTQ+ people have challenges getting their health needs met, including anti-LGBTQ+ stigma in healthcare settings (3–5). Consequently, LGBTQ+ patients may not disclose information about their gender identities, sexual orientations, or comprehensive health needs when seeking care (6) and are potentially less likely to speak out against experiences of prejudice or discrimination when they occur, resulting in suboptimal care or care avoidance (7). Finally, LGBTQ+ people are more likely to be uninsured (8). These challenges may be magnified for LGBTQ+ people who are members of multiple minoritized groups (1, 7).

Primary care is an ideal place to improve LGBTQ+ health due to its person-centered approach, the access it offers to patients of varied social backgrounds, and the prevention, screening, and treatment services it affords to patients across their lifespans (2). Yet, clinics often lack resources to make practice improvements to meet LGBTQ+ patient needs (9, 10). Environmental (e.g., signage, forms) or structural (e.g., policies) elements that exclude LGBTQ+ people may exacerbate feelings of marginalization (9–13). Insensitive, dismissive, or derogatory attitudes and communication from primary care providers (PCPs) and staff can also exert negative impacts (10, 14, 15). A lack of competence in using LGBTQ+ inclusive language, initiating open discussions of health behaviors, and acknowledging patient partners and families (4) can result in negative experiences that reduce satisfaction and retention (14), and quality of care (16, 17). Throughout the U.S., PCPs and staff frequently report a lack of familiarity with, and training on, tailoring services for LGBTQ+ patients (18, 19).

Nationally respected entities, including The Joint Commission, American Academy of Family Physicians, American College of Physicians, Association of American Medical Colleges, and National Academies of of Sciences, Engineering, and Medicine, offer recommendations or guidelines for implementation in healthcare settings to promote equitable LGBTQ+ healthcare (20). These guidelines fall under five categories. The first is LGBTQ+ affirmative policies and procedures, the second is welcoming physical environment, the third is collection of sexual orientation and gender identity (SO/GI) information for patients, the fourth is ongoing training for all employees in LGBTQ+ cultural competency, and the fifth refers to clinical workforce development to encourage delivery of high-quality services for LGBTQ+ patients (1, 15, 20, 21). However, little research has examined the range of individual- and organizational-level factors that influence a clinic's readiness or preparedness to apply guidelines in primary care (2, 15, 22).

Implementing policy and practice changes in primary care is challenged by factors particular to clinics, their employees, and broader healthcare systems (22). Employees may hold differing priorities when implementing guidelines specific to a patient subpopulation, which can diminish buy-in within the organization. Competing demands may also interfere with the implementation of guidelines. Insufficient time or resources can hamper efforts to introduce changes to improve care for patient groups, especially subgroups already stigmatized in healthcare systems (2).

The central question guiding this analysis is: What are the key issues affecting the readiness of primary care clinics to implement guidelines for reducing healthcare inequities for LGBTQ+ people. Readiness can be understood by the R = MC^2^ heuristic: *readiness* (R) is determined by *motivation* (M) of people in the organization to adopt innovations (e.g., guidelines); general organizational *capacities* (C); and innovation-specific *capacities* (C) (23). Motivation includes beliefs about and support for the innovation, both of which contribute to the desire to adopt it. General capacity speaks to aspects of organizational functioning such as culture, climate, staff capacity, and leadership. Innovation-specific capacity describes human, technical, and fiscal conditions such as knowledge, skills, and other innovation-related abilities (23).

## Materials and methods

### Research design

This baseline analysis is part of a multi-phase exploratory/developmental study to advance implementation supports for primary care clinics to initiate or strengthen implementation of national guidelines to enhance healthcare for LGBTQ+ people, including those from diverse racial/ethnic and geographical backgrounds in low-income communities in New Mexico, U.S. (24). For this analysis, we used a concurrent mixed-method research design to identify and analyze individual, organizational, and innovation-specific factors affecting clinics' readiness to implement guidelines before the delivery of implementation supports. This baseline dataset consists of qualitative and quantitative data collected in tandem throughout 2019. The qualitative approach provided the dominant frame for our purposive sampling strategy and analysis (25). The Pacific Institute for Research and Evaluation Institutional Review Board approved the study protocols and informed consent procedures.

The six authors make up our research team. All are white, five are female-identified, five have diverse sexual orientations, and two are genderqueer. Our disciplines span anthropology, medicine, psychology, and sociology. Each has expertise in health services research on reducing disparities for marginalized groups.

### Study context

New Mexico ranks 46th in median household income with the third-largest percentage of residents below the poverty level (18.2%) in the U.S. (26). Hispanic/Latinx and Native American people comprise 60% of residents (27). About 5.1% of adults (28) and 14.5% of high-school students (29) identify as sexual minorities; 0.75% of adults (30) and 3.2% of high-school students (29) identify as gender minorities. The state defines LGBTQ+ people as a protected class of citizens and bans insurance exclusions for transgender people to promote gender-affirming care (31). Nonetheless, access barriers (e.g., stigma and fear) and cultural competence deficits contribute to health and healthcare disparities for LGBTQ+ New Mexicans (14, 32).

### Samples and recruitment

We sought to recruit a minimum of four primary care safety nets or clinics that have historically served patients who are low-income and racially/ethnically diverse regardless of ability to pay (33). A local professional association and our Scientific Advisory Board (SAB) of LGBTQ+ patients, physicians and other providers, healthcare advocates, and researchers assisted with recruitment through email introductions to administrators at federally qualified health centers (FQHCs). An FQHC is a community-based healthcare organization funded by the U.S. Health Services and Resources Administration (HRSA) to deliver primary care services in underserved areas of the country. FQHCs must meet a stringent set of requirements, such as providing care on a sliding fee scale and operating under a governing board that includes patients.

Two researchers met onsite with interested administrators and other personnel at the FQHCs to provide a formal study overview. After this overview, the administrators consulted with PCPs and staff to determine if the clinic would participate. Through this approach, we recruited four primary care clinics (one urban with eight participants; two rural with six and eight participants respectively; one urban/rural with 10 participants). One clinic predominantly served Native Americans, while a second clinic largely cared for immigrant patients of Asian and Hispanic/Latinx origin. All were part of FQHC networks. When the unexpected opportunity arose, we recruited a fifth urban clinic (with four participants) belonging to a university-hospital system based on the SAB's suggestion. The five clinics represented the largest primary care providers in their catchment areas. Two urban clinics had service lines specific to transgender people.

After confirming a clinic's participation, the lead administrators completed a structured collaborative assessment with our researchers to document the presence of LGBTQ+ supportive policies and practices in place in their organization, followed by an in-person qualitative interview. Administrators also facilitated the recruitment of clinic employees, including PCPs (e.g., doctors, physician assistants, nurse practitioners) who deliver medical care and other treatment to patients and frontline staff who fulfill other healthcare roles (e.g., receptionists, medical assistants, outreach workers) for onsite focus groups by advertising the groups at staff meetings, on clinic listservs, and in employee common areas. The inclusion criterion for all participants consisted of working at the clinic for at least 1 year for 20 h per week to ensure familiarity with procedures, practices, and healthcare needs in catchment area populations. The recruitment process resulted in seven individual interviews with administrators; three focus groups with providers/staff (*n* = 5, *n* = 6, *n* = 9) and, because of limited provider/staff availability or interest at the time of data collection, two small group interviews (*n* = 3, *n* = 4) with supplemental individual interviews conducted with clinic providers (*n* = 2). We provided meals at the focus groups and $50 incentives to all participants taking part in a data collection event. Each clinic also received $500 annually for taking part in the implementation component of this exploratory/developmental study.

### Data collection

#### Quantitative surveys

Brief (20-min) hard-copy surveys targeting R = MC^2^ heuristic components were completed immediately before each interview/focus group (23). Table 1 details measures for assessing motivation, general organizational capacity, and innovation-specific capacity.

**Table 1:** Measures included in quantitative data collection.

**Target of R = MC^2^**	**Measure**	**Subscales**	**Number of items**	**Response options**	**Scoring**	**Cronbach's α**
Motivation	Attitudes toward lesbians and gay Men Scale (ATLG) (34)	Attitudes toward lesbians; Attitudes toward gay men	20 items overall; 10 items per subscale	0 = strongly disagree to 4 = strongly agree	Seven reverse coded items recoded so greater scores indicate more negative attitudes; Mean scores per subscale	α = 0.80
	Bisexualities: Indiana Attitudes Scale-Abridged (BIAS-A) (35)	Attitudes toward bisexual men; Attitudes toward bisexual women; Overall BIAS-A	10 items overall; five items per subscale	0 = strongly disagree to 6 = strongly agree	Sum responses within each subscale and subtract # of subscale items answered; Subscale scores combined to generate overall BIAS-A score; Greater scores indicate more negative attitudes	α =0.91
	Attitudes toward Transgender Individuals Scale (ATTI) (36)	Overall ATTI	20 items overall	0 = strongly disagree to 4 = strongly agree	Nine reverse coded items recoded so greater scores indicate more negative attitudes; Mean represents overall attitudes toward transgender individuals.	α = 0.95
	Evidence-Based Practice Attitude Scale (EBPAS) (37)	Openness; appeal	Four items per subscale	0 = not at all to 4 = very great extent	Mean scores per subscale; Greater means indicate more positive attitudes	α_openness_ = 0.78; α_appeal_ = 0.80
General organizational capacity	Organizational Readiness to Change Assessment (ORCA) (38)	Five subscales in the Context module; Staff organizational culture; leadership; measurement; readiness for change; resources	24 items overall	0 = strongly disagree to 4 = strongly agree	Mean scores per subscale; Greater means indicate greater readiness	α_context_ = 0.85; α_stafforgculture_ = 0.90; α_leadership_ = 0.93; α_measurement_ = 88; α_readiness_ = 0.91; α_resources_ = 0.86
Innovation-specific capacity	Lesbian, Gay, Bisexual, and Transgender Development of Clinical Skills Scale (LGBT-DOCSS) (39)	Clinical preparedness; Attitudinal awareness; Basic knowledge	18 items overall	0 = strongly disagree to 6 = strongly agree	Eight reverse coded items recoded; Mean ratings per subscale; Greater means represent greater clinical preparedness, attitudinal awareness, and basic knowledge	α_overall_ = 0.86; α_clinprep_ = 0.88; α_attaware_ = 0.80; α_know_ = 0.83
	Implementation Climate Scale (ICS) (40)	Focus; Educational support; Recognition; Selection for evidence-based guidelines (EBG); Selection for general openness	15 items overall; three items per subscale	0 = not at all to 4 = very great extent	Mean ratings for each subscale indicate a more positive climate for implementing EBG	α_overall_ = 0.91; α_focus_ = 0.91; α_edusupt_ = 0.84; α_recog_ = 0.88; α_selectEBG_ = 0.89; α_selectopen_ = 0.91

##### Motivation

Four measures explored motivation, including the *Attitudes toward Lesbians and Gay Men Scale* (ATLG; α = 0.80) (34), *Bisexualities: Indiana Attitudes Scale-Abridged* (BIAS-A; α = 0.91) (35), *Attitudes toward Transgender Individuals Scale* (ATTI; α = 0.95) (36), and the *Evidence-Based Practice Attitude Scale* (EBPAS) (37). The EBPAS Openness and Appeal subscales were explored, referring to a general willingness to try new practices and the positive perception of the new practices, respectively.

##### General organizational capacity

We administered five subscales in the Context module (α = 0.85) of the *Organizational Readiness to Change Assessment* (ORCA): one dimension on staff organizational culture, the leadership subscale on formal leadership and teambuilding, the measurement subscale on evaluation of goal setting and tracking and communicating performance, the readiness for change subscale on the attitudes of opinion leaders for practice change, and the resources subscale centering on availability of funds, staff time, facilities, and equipment to support changes in general (α = 0.86) (38).

##### Innovation-specific capacity

Two measures included the *Lesbian, Gay, Bisexual, and Transgender Development of Clinical Skills Scale* (LGBT-DOCSS; α = 0.86) (39) and the *Implementation Climate Scale* (ICS; α = 0.91) (40).

#### Structured collaborative assessment

We developed a comprehensive checklist based on criteria from nationally respected entities to assess current levels of implementation of (1) LGBTQ+ affirmative policies and procedures, (2) physical environment, (3) SO/GI data collection, (4) training, and (5) clinical workforce development at each site (20, 41–43). The SAC was completed together by two researchers and clinic administrators with a visual inspection by the former and supporting documentation provided by the latter.

#### Qualitative interviews/focus groups

We developed semi-structured interview and focus group guides containing questions related to the organizational attributes of clinics and attitudinal factors, behaviors, and experiences related to care for LGBTQ+ people. The questions were informed by a review of national guidelines and consultation with the SAB. Accordingly, the questions centered on general knowledge of and experience with LGBTQ+ patients, facilitators/barriers to using the LGBTQ+ inclusive guidelines, and factors likely to affect both readiness and implementation. The 60- to 90-min interviews/focus groups were digitally recorded, transcribed, and reviewed for accuracy.

### Data analysis

#### Quantitative analysis

We calculated total and subscale scores and assessed descriptive statistics for central tendency, variability, and skewness. Paired-sample *t*-tests and general linear modeling in SPSS Version 25 assessed between-subscale differences controlling for within-rater agreement. When between-subscale differences were found, follow-up tests using orthogonal contrast codes were examined to better understand the pattern of between-subscale differences. Due to the modest sample size, all analyses were conducted across the full sample, and between-participant comparisons in total and subscale scores were not examined.

#### Qualitative analysis

We used a question-level coding process to analyze textual data, which involved iterative coding, analysis, writing, revision, and use of an Excel spreadsheet to organize and manage the data. Two analysts (including the first author) began by developing a deductive coding structure featuring codes derived from the topic areas and questions embedded in the data collection guides (e.g., education in LGBTQ+ competent care, addressing racial/ethnic diversity). We also incorporated key sensitizing concepts from the implementation science literature (e.g., openness to innovation, leadership) into this structure (25). We reviewed each transcript, assigning codes to segments of text ranging from a phrase to several paragraphs. Open coding was used to identify and define new codes related to ideas that we had not previously considered (e.g., silo effect, lack of behavioral healthcare), followed by focused coding to determine which ideas recurred or represented unique participant concerns (44). The larger team compared and contrasted codes during its regular meetings, grouping similar content or meaning into broader themes, creating an outline to describe linkages, and drawing on the SAC tabulations of current implementation levels of LGBTQ+ inclusive policies and practices for additional context (25, 44). We shared summaries of key findings with the SAB *via* PowerPoint presentations for collective discussion and interpretation.

#### Mixed-method analysis

We created matrices to triangulate quantitative and qualitative data and assess: (1) convergence (the extent to which the quantitative and qualitative results share similar findings); (2) expansion (the degree to which findings of one dataset are explained by the other); and (3) complementarity (the contextualization of results by embedding findings from one dataset into the other) (24, 25). The R = MC^2^ heuristic guided the identification of factors likely to impact readiness to implement LGBTQ+ inclusive guidelines. To aid our interpretation of findings we also turned to political philosopher Iris Young's theory of structural injustice, which is useful for problematizing commonplace, often unchallenged social processes and institutional routines that render certain social groups vulnerable to domination or oppression (45, 46).

## Results

### Sample

Two of the 36 participants did not complete the survey. During qualitative data collection, these two individuals self-identified as cisgender males, heterosexual, and of Hispanic/Latino origin. See Table 2 for available demographic information from the survey.

**Table 2:** Participant demographics based on survey responses.

**Variable**	***n***	**%**
Gender identity or expression^*^		
Female	24	75
Male	8	25
Missing/not reported	2	
Sex assigned at birth^**^		
Female	26	76.5
Male	8	23.5
**Race**		
American Indian, Alaska Native, Indigenous Latin American	4	11.8
Asian American or Asian	1	2.9
European American, White or Anglo	25	73.5
Missing/Not reported	4	
**Ethnicity**		
Non-hispanic	17	50
Hispanic	17	50
**Sexual orientation**		
Bisexual	2	6.7
Heterosexual	21	70
Lesbian/gay	6	20
Queer	1	3.3
Missing/not reported	4	
**Highest level of education**		
Completed high school or GED	3	8.8
Completed trade/vocational school	3	8.8
Some college, no degree	4	11.8
Completed Associates degree	6	17.6
Completed Bachelor's degree	3	8.8
Some graduate school, no degree	1	2.9
Completed graduate/professional	14	41.2
**Variables**	$\bar{x}$ **±** sd	
Age (years)	45.97 ± 12.6
Years at FQHC	6.50 ± 6.3
Years in primary care	12.73 ± 10.1
Years in urban primary care	1.45 ± 0.5

* For Current Gender Identity or Expression, participants could select multiple options from the following list: female; male; transgender man/transman; transgender woman/transwoman; genderqueer/gender non-conforming; other (specify); and prefer not to answer.

** For sex assigned at birth, participants could select one option from the following list: female; male; intersex; other (specify); and prefer not to answer.

### Quantitative results

Table 3 describes the results of quantitative analyses for this baseline study. When exploring motivation, there were no significant differences in attitudes toward lesbians and gay men or attitudes toward female bisexuals and male bisexuals. Attitudes toward transgender people were generally positive. Openness toward, and the intuitive appeal of, evidence-based guidelines were similarly positive.

**Table 3:** Descriptive and inferential statistics from quantitative survey measures.

**Target of R = MC^2^**	**Measure**	**Subscale**	**$\bar{\text{x}}$, *sd***	**Test of between-subscale differences**
Motivation	ATLG	Lesbians	0.59, 0.56	Lesbian vs. gay men[*t*_(32)_ = 0.712, *p* = 0.482]
		Gay men	0.52, 0.61	
	BIAS-A	Female bisexual	2.33, 4.6	Female vs. male bisexual [*t*_(32)_ = 0.821, *p* = 0.418]
		Male bisexual	2.09, 4.32	
	ATTI	Overall attitudes	0.42, 0.58	Not applicable
	EBPAS	Openness	3.50, 0.82	Openness vs. appeal [*t*_(32)_ = 0.154, *p* = 0.878]
		Appeal	3.48, 0.72	
General organizational capacity	ORCA	Readiness for change	3.37, 0.76	Readiness vs. all else [*F*_(1, 32)_ = 14.29, *p* = 0.001]; Staff organizational culture vs. leadership, measurement, and resources [*F*_(1, 32)_ = 14.94, *p* = 0.001]; Measurement vs. resources [*F*_(1, 32)_ = 14.68, *p* = 0.001]
		Staff organizational culture	3.24, 0.77	
		Leadership	3.11, 1.12	
		Measurement	2.99, 0.98	
		Resources	2.39, 1.10	
Innovation-specific capacity	LGBT-DOCSS	Attitudinal awareness	5.52, 0.78	Attitudinal awareness vs. all else [*F*_(1, 32)_ = 35.51, *p* < 0.001]; Knowledge vs. preparedness [*F*_(1, 31)_ = 0.01, *p* = 0.936]
		Basic knowledge	4.00, 1.72	
		Clinical preparedness	3.87, 1.70	
	ICS	Focus	3.42, 0.91	Focus vs. all else [*F*_(1, 30)_ = 31.40, *p* < 0.001]; Selection for openness vs. educational support, recognition, and selection for EBP [*F*_(1, 30)_ = 11.36, *p =* 0.002]
		Selection for openness	3.22, 1.07	
		Educational support	2.52, 1.22	
		Recognition	2.48, 1.19	
		Selection for EBG	1.52, 1.32	

When exploring general organizational capacity, the highest ratings were for the readiness for change subscale targeting the attitudes of opinion leaders for practice change. Staff organizational culture, leadership, measurement, and resources to support changes followed. Follow-up contrast codes indicated readiness scores were significantly greater than all other subscales. Staff culture was rated significantly higher than leadership and teambuilding, measurement, and resources. Leadership and teambuilding were significantly higher than measurement and resources. Lastly, measurement was significantly higher than resources.

The LGBT-DOCSS and ICS explored innovation-specific capacity. Results suggested greater attitudinal awareness than basic knowledge or clinical preparedness combined. There was no significant difference between basic knowledge and clinical preparedness. Implementation climate for LGBTQ+ inclusive policies and practices varied. The focus subscale was rated the greatest, followed by selection for general openness, educational support, recognition, and selection specifically for expertise/experience with LGBTQ+ inclusive policies and practices. Follow-up contrast comparisons revealed significantly higher ratings on the focus subscale than all others, and significantly higher ratings for selection for general openness than educational support, recognition, and selection specifically for expertise/experience with inclusive policies and practices combined.

### Qualitative results

Iterative coding and analysis processes resulted in six themes: treating all patients the same; addressing diversity in and across LGBTQ+ populations; clinic climates; patient access concerns; insufficient implementation support; and leadership considerations. We include quotations representing the perceptions and experiences of participants for each theme, and integrated SAC tabulations in our presentation of findings. Table 4 includes a summary of each theme, along with key points and representative quotes.

**Table 4:** Overview of qualitative results.

**Theme**	**Representative quotes**	**Key points**
“Treating all patients the same”	“As an FQHC, we don't turn away anybody. It doesn't matter sex, gender, [or] financial status.” (Nurse practitioner) “I don't see any difference from [LGBTQ+ patients than] from other everyday people.” (Medical assistant) “That's something I don't think about—of what their thoughts are regarding LGBTQ. I see them as a patient. I take care of them and make sure that they're doing well.” (Physician) “We're an all-inclusive clinic…. Our mission, vision, and values clearly state it.” (Administrator)	1. In the absence of formal medical training, relying on interpersonal skills to care for LGBTQ+ patients becomes the main strategy for care delivery. 2. Clinic personnel desire more structured training experiences in lieu of hands-on or virtual learning. 3. Mission-driven and values-based care can be a motivator, yet it can also detract from providing equitable care to LGBTQ+ patients.
Addressing diversity in and across LGBTQ+ populations	“It's something that is not accepted. Grandma and grandpa, mom and dad, it's just not…in the Hispanic culture…we're very private about stuff like that.” (Administrator) “It is difficult in a clinic…with lots of different backgrounds and cultural views, how to figure out the best way.” (Physician) “The translators we have, they are fluent in the Spanish. But there're different Spanish and different words. Sometimes it's like, ‘How do you say that word?” (Administrator)	1.Cultural considerations on the part of patients and clinical personnel can elide discussions of sexuality and gender identity during care delivery. 2. Navigating intersections of when, how, and with whom to discuss sexual orientation and gender identity can be challenging, even for people trained in LGBTQ+ healthcare. 3. Translation options, including digital options, are poor and of little use in this area.
Clinic climates	“They feel ashamed of going to the doctor and having that stigma toward them.” (Physician) “I can't think of anything that's either super heteronormative or not… I'll say our environment is probably neutral. It's not too bad either way.” (Administrator) “I would actually like to see more questions on there about stuff like, ‘Were you made to feel uncomfortable at all about your income and your insurance? Were you made to feel uncomfortable about your gender identity?” (Physician)	1.While the importance of climate had tangible impacts, making recommended climate changes is perceived as challenging. 2. Participants reference bias at the provider/staff level, fear and stigma among patients, lax or unenforced anti-discrimination policies, and non-inclusive physical environments. 3. Clinic personnel are enthusiastic about ideas for improving climate to better support LGBTQ+ patients, and believe patient feedback can be important to enhance climates.
Patient access concerns	“I think in [name of locality], a lot of people in the healthcare community historically have just said, ‘Oh, we don't really need to worry about that because [name of clinic] does it” (Administrator) “We don't have any experience with it. We're not getting any experience with it. None.” (Outreach worker)	1.Specializing in care can increase access and visibility, but reduce the incentive to ensure everyone is capable of delivering LGBTQ+ competent care. 2. In rural areas, the lack of focused services requires patients to utilize more resources to get necessary care.
Insufficient implementation support	“Everyone is so overwhelmed. We're resource-poor, timewise, like in every way, and asking [staff] to do more things is a lot to ask.” (Physician) “When we do these trainings, they have to be time-limited because we can't have them open-ended…and we have about 200 employees.” (Administrator) “Our EHR does not allow for us to put the name in the patient chart. We have to put it in a sticky and hope that whoever is going to call them, calls them by the name they want to be called by...” (Medical assistant)	1.Increasing implementation responsibility for already overworked people at underfunded clinics is a barrier to successful organizational change. 2. Targeted, time-limited onsite training is a feasible option to engage clinic personnel in continued education. 3. The collection and subsequent use of SO/GI data are problematic for clinics, regardless of whether EHRs are configured to document this information.
Leadership considerations	“Our funding is contingent on whether or not we follow their guidelines.” (Administrator) “The feds do have a lot of power in both a bad way and a good way, but they could be doing a lot more to promote it [inclusive primary care for LGBTQ+ people].” (Administrator)	1.Funding sources exert influence over the implementation priorities of clinics. 2.Greater top-down incentives (including from federal sources) may improve motivation and capacities for LGBTQ+ inclusive primary care

#### Treating all patients the same

Participants at all clinics noted the diversity of patients their clinics cared for. One physician observed, “We have ages from zero to 100…. Not only are [our patients] culturally diverse, but we also need to understand our youth, and older patients, and families, and try to get all those different groups recognized…that's sometimes hard.” Caring for everyone meant not prioritizing one segment of the patient population. Echoing the viewpoints of other fellow participants, a nurse practitioner explained, “As an FQHC, we don't turn away anybody. It doesn't matter sex, gender, [or] financial status.”

While describing an openness to working with LGBTQ+ patients, many participants admittedly lacked a depth of understanding of LGBTQ+ competent care practices. One physician said, “I've been a doc for over 30 years, and I still feel it's an area that I'd like to know more about.” PCPs faulted medical education for insufficient LGBTQ+ training opportunities and claimed it was up to them to acquire training to better prepare them to work with LGBTQ+ patients. For example, a second physician disclosed that when asked to direct a service line for transgender patients, they had no training in caring for the population and relied heavily on the internet to obtain clinical guidance.

The SAC affirmed that clinics did not require providers/staff to take part in LGBTQ+ cultural competency training on an ongoing (e.g., annual) basis, and only one clinic provided training on such topics as part of its new employee orientation process. One clinic employed providers specifically trained in treating conditions common among LGBTQ+ patients. Nonetheless, participants voiced a great deal of confidence that their LGBTQ+ patients would receive quality care predicated on convictions of “treating all patients the same.” One medical assistant stated, “I don't see any difference from [LGBTQ+ patients than] from other everyday people.” A physician emphasized, “That's something I don't think about—of what their thoughts are regarding LGBTQ. I see them as a patient. I take care of them and make sure that they're doing well.” Relatedly, participants noted that services were “mission-driven,” with an administrator emphasizing “We're an all-inclusive clinic…. Our mission, vision, and values clearly state it.”

#### Addressing diversity in and across LGBTQ+ populations

Participants pointed to several cultural factors, including age, upbringing, and language, affecting primary care and help seeking among LGBTQ+ patients. Participants serving rural, less socially liberal areas reported discomfort asking older patients SO/GI questions and attributed this to their own cultural upbringings. One administrator explained, “My parents were very conservative. They didn't talk about those kinds of things…. I never had that exposure.” Unfamiliar terms used by younger LGBTQ+ people similarly posed a challenge for clinic personnel, as did assuring patient confidentiality for LGBTQ+ youth. A medical assistant explained, “It's so hard to ask youth questions. I can recall one incident. I felt it wasn't right, so I separated dad from son…. [I] asked him, and he said, ‘I'm gay and I cannot tell my dad. He'll kill me.”'

Participants struggled to discuss differences between and among LGBTQ+ populations of various racial/ethnic backgrounds and how these differences affected patient needs. Responses centered on Hispanic/Latinx people who participants categorized as “traditional,” citing the role of Catholicism and fundamentalist churches in shaping attitudes about LGBTQ+ people. A parent of two LGBTQ+ children and a medical assistant at a rural clinic explained, “It's something that is not accepted. Grandma and grandpa, mom and dad, it's just not…in the Hispanic culture…we're very private about stuff like that.” Per the SAC, only two clinics reportedly had PCPs who were trained in taking LGBTQ+ inclusive health histories. One such physician underscored the complications involved with patients who did not respond well to questions about their gender and sexuality, reflecting on a recent encounter with a patient from the Democratic Republic of Congo who “clammed up” when asked such questions. Concerned that the questions were “culturally insensitive,” this physician disclosed, “It is difficult in a clinic…with lots of different backgrounds and cultural views, how to figure out the best way.”

Language barriers amplified complexities with non-English speaking patients. An administrator explained, “The translators we have, they are fluent in the Spanish. But there's different Spanish and different words. Sometimes it's like, ‘How do you say that word?”' This problem was pronounced in clinics serving immigrant and refugee populations, including those who spoke languages other than Spanish. Here, clinicians in cross-cultural medical encounters relied on language translation apps or devices that were insensitive to LGBTQ+ terminology.

#### Clinic climates

In describing climates, participants referenced bias at the provider/staff level, fear and stigma among patients, lax or unenforced anti-discrimination policies, and non-inclusive physical environments. They indicated that patient feedback can help enhance climates.

Participants across clinics referenced coworkers whose prejudicial attitudes and behaviors could contribute to a negative climate. One administrator explained, “People are difficult in terms of changing their views or ways, so we try to heighten their sensitivities and implement procedures that require their compliance at least.” Participants believed such persons comprised a minority of staff. At the same time, however, they suggested that fears about how clinic personnel will perceive and treat them led LGBTQ+ patients to delay or avoid care. One physician explained, “They feel ashamed of going to the doctor and having that stigma toward them.” Participants also surmised that apprehension about confidentiality breaches, i.e., clinic personnel gossiping about the LGBTQ+ status of patients, curtailed help seeking, speculating that a clinic's climate could dissuade patients from being open about their identities. A nurse practitioner noted that, in contrast to heterosexual patients, few LGBTQ+ patients introduce “their partner as their partner.” They added, “That tells me there's a culture of secrecy or there's a culture of not feeling comfortable coming into a clinic.”

Participants at one rural clinic critiqued its patient non-discrimination policy for not stipulating LGBTQ+ protections. This publicly posted policy did not identify any category of persons warranting special protections. Three clinics already had policies prohibiting discrimination based on gender identity or sexuality per the SAC. Still, participants within these settings suggested that awareness and enforcement of such policies were lax.

In general, the décor of clinics was not inclusive of LGBTQ+ people. An administrator explained, “I can't think of anything that's either super heteronormative or not… I'll say our environment is probably neutral. It's not too bad either way.” Per the SAC, only one clinic had LGBTQ+ inclusive posters, pictures, symbols, or flags visibly displayed upon entrance.

A second clinic posted pictures of culturally diverse people of color in public waiting areas, displaying PRIDE and Safe Zone stickers in far less visible spaces, while a third clinic was barred from posting any items on its walls. LGBTQ+ inclusive patient education materials were only available in the two clinics with gender minority service lines.

Patient access to gender-inclusive restrooms was variable across clinics. The SAC documented that four clinics had multi-stall restrooms marked by gender in public waiting areas. However, none posted signs indicating patients' right to choose the most suitable accommodation, nor statements to request access to single-stall restrooms. The fifth clinic was transitioning its single-stall restrooms in the waiting area but was delayed due to ordering signage signifying gender-inclusiveness. Nevertheless, participants from all clinics enthusiastically endorsed transitioning to gender-inclusive restrooms and posting relevant signage to support a welcoming environment.

The SAC affirmed that patient feedback surveys distributed by the five clinics did not collect demographic data, including identification as an LGBTQ+ respondent. Yet, participants proposed patient satisfaction surveys as one way to learn about experiences of service provision from LGBTQ+ patient perspectives. In one small group interview, a physician explained, “I would actually like to see more questions on there about stuff like, ‘Were you made to feel uncomfortable at all about your income and your insurance? Were you made to feel uncomfortable about your gender identity?”' The group discussed that integrating such questions would generate important feedback for improving the clinic's climate for LGBTQ+ patients and other marginalized groups.

#### Patient access concerns

Participants in all clinics raised concerns about limited support and services for LGBTQ+ patients. One clinic maintained a list of community providers and specialists supportive of LGBTQ+ patients for referrals. Yet, most participants lacked detailed knowledge of available supports and services and how they could be accessed. Participants from three clinics reported care as being “siloed” in the community and their workplaces. For example, an administrator explained how another agency in the community had established a reputation for caring for persons with HIV in the 1990s, which filled a niche and reduced incentive for other clinics to develop capacity in caring for the LGBTQ+ population. The administrator stated, “People in the healthcare community have just said, ‘Oh, we don't really need to worry about that because [name of clinic] does it.” This administrator's clinic referred HIV-related clinical matters to the neighboring agency instead of building its in-house capacity. Not all patients referred to the other clinic for Pre-Exposure Prophylaxis (PrEP) were said to be satisfied with this arrangement, wanting to exert choice over where they received care.

Participants debated whether patients should be channeled to specific PCPs within an organization. Such PCPs could be a resource, particularly if other PCPs were uncomfortable addressing LGBTQ+ considerations. However, participants warned that this could engender a silo effect within the organization itself. For example, in the two clinics with specialty services for gender minority patients, participants expressed concern that their colleagues were less inclined to bolster their own knowledge and skills because they had coworkers to depend on. This resulted in a separation between gender minority and cisgender patients in clinicians' caseloads. Because the specialty services were in high demand, these participants wanted to diffuse appropriate knowledge and skills across the clinical workforce, arguing that gender-affirming care did not always require specialized medical knowledge. Participants in the three other clinics could only identify one to two clinicians, if any, who delivered hormone therapy, PrEP, or post-exposure prophylaxis (PEP). There were no PCPs on staff well-practiced in hormone therapy for gender-affirming care in the two rural clinics. The imperative to develop this capacity among staff was low, with an administrator stating, “We don't have a lot of folks coming in asking for hormonal treatment stuff…. It's not something that's come up as, ‘Oh, we had to tell this person we couldn't care for them here or something like that.”'

Participants reported a lack of referral resources and specialty services for LGBTQ+ patients, particularly for behavioral healthcare. There was broad consensus among participants serving rural areas that some patients struggling with behavioral health concerns would benefit from support beyond what clinics could provide, such as support groups oriented toward LGBTQ+ people. However, participants were unaware of such groups that might be helpful to LGBTQ+ people. Travel was a frequently mentioned barrier to care. An “out” outreach worker had knowledge of LGBTQ+ behavioral health services in a community 60 miles from the clinic but believed that a lack of transportation prevented most LGBTQ+ patients from using these services. Without in-house expertise or readily identifiable places locally, patients had little choice but to incur time and transportation expenses to obtain services elsewhere.

Participants suggested that some access barriers could be reduced through outreach to LGBTQ+ patients, including advertising directly to LGBTQ+ patients. One administrator described their clinic as being “open” because it provided HIV prevention, testing, and treatment services. However, because the clinic did not directly advertise its services to LGBTQ+ people, patients were unlikely to know that “this organization is LGBTQ+ friendly” and consequently be “scared that the service provider would treat someone different or with disrespect for coming into this organization for being who they are.” Of note, three clinics had histories of staffing booths at local PRIDE fairs; however, they distributed only basic information about services and programs for the general public without marketing tailored to LGBTQ+ people. Booths were primarily staffed by LGBTQ+ or allied employees.

#### Insufficient implementation support

Participants reported a lack of implementation support for advancing improvements in LGBTQ+ primary care. These included demanding workday realities, mandates without training, and technological constraints.

##### Workday realities

Participants described time and resource constraints, coupled with a lack of engaged champions, which reduced capacity to enhance services for LGBTQ+ people. Staff were pressured to see a high volume of patients in a short period, making it hard to implement new practices. A physician clarified, “Everyone is so overwhelmed. We're resource-poor, timewise, like in every way, and asking [staff] to do more things is a lot to ask.” Such constraints meant PCPs and staff were disinclined to invest much time and effort into implementing guidelines for improving care for LGBTQ+ patients, even if they supported such change. This physician continued, “It's a want [the improvements]. It's on the wish list, but it's just not high, and [clinic personnel are] not forced to, or there's not time specifically carved out…and they have no personal pull toward [it].”

##### Training

Participants across clinics endorsed training staff in LGBTQ+ topics, particularly around collecting SO/GI data. However, frontline staff were uneasy asking patients about their sex assigned at birth and current gender identities. The four FQHC clinics funded by HRSA were mandated to report gender identity data only. To comply, leaders added queries to registration forms and instructed frontline staff to obtain this information, yet there was no associated training. Hence, staff were unfamiliar with acquiring the information, and some feared disrespecting or annoying patients. Without training on gathering such information, staff reportedly marked a patient's gender identity based on their own impressions and did not directly ask or encourage patients to answer SO/GI queries. In the fifth clinic where such training had occurred, turnover created challenges for ensuring that frontline staff were consistently prepared.

Most participants suggested that they and their colleagues would benefit from education in LGBTQ+ healthcare practices. They were interested in honing their soft skills for interacting with LGBTQ+ patients, learning best practices for eliciting SO/GI information or applying appropriate pronouns and terminology (two topics returned to below). Such training was considered relevant to all staff. Citing the gaps in their medical education (described above), PCPs wanted to learn more about best practices for caregiving with LGBTQ+ patients but suggested that time constraints and competing demands prevent this from happening.

Limited LGBTQ+ training opportunities were available onsite. There was broad consensus that training was best provided in small groups to promote engagement and reduce distraction. “You don't feel so rushed, and maybe…you feel more focused,” stated one medical assistant. Regardless of format, training took time away from the performance of other work duties that generated billable hours, as an administrator claimed, “When we do these trainings, they have to be time-limited because we can't have them open-ended…and we have about 200 employees.”

##### Technological constraints

Participants across clinics called attention to deficiencies in electronic health records (EHR) systems for documenting and using SO/GI data in patient care encounters. Per the SAC, three clinics collected SO/GI in the EHR. The other two collected gender identity only. However, information on chosen name and pronoun usage was not inputted in standardized ways but instead delegated to fields for clinical notes that were not searchable or accessed widely. A medical assistant observed, “Our EHR does not allow for us to put the name in the patient chart. We have to put it in a sticky and hope that whoever is going to call them, calls them by the name they want to be called by...” Across clinics, “sticky notes” emerged as the most common way to flag gender-diverse patients.

#### Leadership considerations

Participants characterized leadership support to move forward with LGBTQ+ healthcare guidelines as high and across all levels. One administrator characterized “supervisor and manager buy-in” as critical to a successful rollout of such guidelines. An administrator remarked, “I'd really be shocked if they came back and told me they didn't want to do it.” Yet this sentiment rarely manifested through tangible supports for innovation. Most administrators and leaders interviewed also lacked in-depth knowledge of guidelines or best practices for improving care for LGBTQ+ patients. Those with such awareness recognized challenges in their daily work that prevented action (i.e., being pulled in multiple directions and limited resources). Such participants also described efforts to initiate change (e.g., reaching out to local LGBTQ+ organizations, distributing patient surveys), yet reported time/resource constraints hindering follow-through on such efforts.

Participants suggested that governance bodies (e.g., Board of Directors) might benefit from targeted education related to caring for LGBTQ+ people and that HRSA regulations were pivotal at the organizational level for building a clinic's capacity to pursue innovations. One administrator observed, “Our funding is contingent on whether or not we follow their guidelines.” Underscoring this point, another administrator stated, “The feds do have a lot of power in both a bad way and a good way, but they could be doing a lot more to promote it [inclusive primary care for LGBTQ+ people].” Unless mandated by HRSA to take on new initiatives, administrators were less inclined to actively pursue such practice-level changes organizationally. A third administrator explained, “When it comes to something extra…the way to get it to move to the front of the line is to get HRSA to recommend it or mandate it…[and]…to get it included in the UDS [Uniform Data System] Clinical Quality Measures.” This administrator asserted that clinics had “a long list of things we have to report,” and lacked “a huge amount of bandwidth to do a lot of other things.”

## Mixed-method results

Our concurrent mixed-method approach demonstrated convergence, expansion, and complementarity between the two types of data collected from the same participants (24, 25). Findings from both types provide insight into the readiness of primary care clinics to improve service environments and implement inclusive policies and practices for LGBTQ+ patients based on national guidelines. The combined results affirm there is room for improvement in implementation climate and organizational readiness for change to reduce access barriers and enhance high-quality services for LGBTQ+ patients.

In terms of motivation at the individual level, quantitative findings illustrate accepting attitudes about LGBTQ+ people among our purposive sample of administrators, providers, and staff. Openness toward and the intuitive appeal of LGBTQ+ inclusive guidelines were also very positively rated. The qualitative results elaborated these findings, with participants expressing both interest in and support for changing service environments, policies, and practices to improve the experience of LGBTQ+ patients. However, they suggested that motivation could be undermined by a perceived lack of demand for LGBTQ+ responsive services or personal connection to LGBTQ+ people, concern about making heterosexual and cisgender patients uneasy, and the siloing of services. Cultural factors, i.e., the social backgrounds of staff and small town/rural community contexts, could also hamper motivation. Sensing the presence of stigma and fear in their clinic climates, participants indicated that such cultural factors could compromise care by leading providers/staff to avoid asking inclusive questions about gender and sexuality or seeking permission from patients to ask such questions.

The quantitative and qualitative findings concerning general capacity at the organizational level were mixed. Quantitatively, participants positively rated attitudes of opinion leaders, staff organizational culture, formal leadership, and teambuilding as supportive of the implementation of LGBTQ+ inclusive policies and practices. However, measurement of goal setting and tracking and communicating performance were rated less favorably, while resources to support changes were rated least favorably. The qualitative data uncovered rules prohibiting the posting of imagery to signify inclusivity of any patient population, a possible lack of engaged champions, and inadequate material resources to enable implementation, i.e., capital for education, gender-affirming restrooms, or EHR modifications. Other pragmatic considerations, such as a high volume of diverse patients and overwhelmed staff, challenged the justification for improving services for specific populations. Constraints on general organizational capacity also limited the ability of clinics to create welcoming environments or conduct targeted community-based outreach to LGTBQ+ patients. Having to respond to federal mandates for funding deterred leaders from investing time and resources in new initiatives unrelated to these mandates. These same constraints were potentially aggravated by a lack of behavioral healthcare and other supportive resources in communities, exacerbating access challenges for LGBTQ+ patients.

Results regarding innovation-specific capacity were likewise mixed. Both quantitatively and qualitatively, participants reported greater awareness or focus within their clinics for prioritizing the implementation of LGBTQ+ innovations. However, our participants were less likely to report actual behaviors and skills related to clinical care for LGBTQ+ patients at the individual level or selection, recognition, or educational support actions to facilitate implementation at the organizational level. Qualitatively, they described treating LGBTQ+ patients the same as all other patients to reduce health disparities yet simultaneously affirmed the need for education to nurture general LGBTQ+ competency (e.g., language use, SO/GI data collection, addressing privacy/confidentiality concerns) and to diffuse knowledge in clinical care (e.g., gender-affirming hormone therapy). These results also emphasized gaps in human, technical, and fiscal conditions for nurturing innovation-specific capacities.

## Discussion

Primary care clinics are responsible for delivering equitable care to all patients, including persons of diverse gender identities and sexual orientations (21). Benefits to improving care for LGBTQ+ patients include reducing healthcare disparities, service costs, and illness progression and transmission, and enhancing mental and physical wellbeing and longevity. This study affirms the need to address readiness among clinics and their personnel to enable the implementation and sustainment of established guidelines for achieving health equity for LGBTQ+ people. It also demonstrates the practical applicability of the R = MC^2^ heuristic for examining readiness at individual and organizational levels to implement the guidelines (23). Overall, participants expressed great motivation for improving care for LGBTQ+ patients, although they reported insufficient organizational and innovation-specific capacities to do so.

This study highlights an overarching and problematic perceived imperative to “treat all patients the same.” This imperative, predicated on ideologies of turning no patients away, appeared fundamental to care ethics and commitments to serving diverse populations of varying racial/ethnic, cultural, and geographical backgrounds in this study's clinics. Participants commonly cited such messaging to convey their support for equal healthcare for all. However, instantiated within clinic mission statements and policies, this shared attitude toward LGBTQ+ patients in primary care may also promote institutional structures that marginalize LGBTQ+ patients and their families and undermine staff and provider motivation to change behaviors, processes, and practices that are potentially harmful to LGBTQ+ patients (15, 32).

Iris Young's theory of structural injustice provides a useful framework for demonstrating how a multiplicity of unjust social processes and factors reinforce one another and become the status quo, creating and maintaining social inequalities in systems such as primary care (45). Structural injustice, in this case the omission of LGBTQ+ affirmative healthcare practices under the auspices of “treating patients the same,” places LGBTQ+ patients in clinical encounters in which they are cared for in ways befitting the standard or dominant patient, i.e., one who is White, English-speaking, heterosexual, and cisgender. This approach affectively negates the socially determined experiences of underrepresented patients, yet is not dependent on the purposeful wrongdoing of individual providers or staff. It results instead “as a consequence of many individuals and institutions acting in pursuit of their particular goals and interests, within given institutional rules and accepted norms” (45). Hence, well-intentioned individuals maintain structural injustices and perpetuate unequal healthcare treatment *via* “unquestioned norms, habits, and symbols, in the assumptions of underlying institutional rules and the collective consequences of following those rules” (46). Disrupting these missions and messages is crucial, as they reduce the readiness to adopt LGBTQ+ inclusive guidelines by enabling PCPs and staff to neglect their own implicit biases or unintentional discrimination, resulting in disinterest in pursuing organizational change and the “cultures of secrecy” described by participants.

Nevertheless, there are several actions clinics can take to overcome the inertia that can thwart the implementation of LGBTQ+ inclusive guidelines. One first step is developing an implementation team of providers and staff who can serve as champions and meet regularly to assess readiness needs at the organizational and individual levels and plan to adopt and maintain the guidelines (47). To ensure success, team members should be provided with protected time, acknowledged, and rewarded by clinic leadership for taking on this work, thus conveying broader organizational support for guideline implementation (20). Alignment across a clinic's leadership at multiple levels (e.g., executive, middle management, supervisory) is also likely to be critical to effective implementation. Leaders can set expectations for change and promote buy-in by becoming knowledgeable about the guidelines, talking up the guidelines in the clinic, allocating resources, problem-solving with the implementation team to overcome challenges, and celebrating successes (48).

Organizational assessments can illuminate a clinic's current state of readiness, including key facilitators (e.g., current policies, the influence of key opinion leaders) and barriers to be fixed or modified (e.g., time limitations, siloing effects). They can also be used to identify invisible barriers that sustain seemingly innocuous policies that actually serve to marginalize and silence non-normative patients, such as those who are gender or sexual minorities (45, 49). Data for assessments can come from multiple sources (e.g., EHRs, written documents, surveys and focus groups with patients, providers, and staff) and inform the creation of action plans or blueprints to guide implementation (20, 22). By attending to implementation environments and diverse stakeholder needs, such assessments can help determine acceptable, appropriate, and feasible implementation strategies or methods for enhancing the likelihood that an innovation will be implemented successfully (47, 50). Because readiness is likely to shift over time due to factors described in our analysis (e.g., turnover, productivity requirements), assessments should be repeated regularly in order to determine progress made toward LGBTQ+ health equity improvements and update action plans (23).

Education that promotes knowledge about LGBTQ+ inclusive policies and practices (as well as cultural humility to counter neutralizing discourses about treating all patients the same) is a critical implementation strategy, not only for facilitating the adoption of new guidelines, but for catalyzing shifts from the rote provision of healthcare to open conversations about patients' lived realities and care needs. Education in the form of one-shot or infrequent didactic trainings is unlikely to produce sustainable change in primary care practice. Education should instead become embedded in the workplace, occurring on an ongoing (e.g., annual) basis beginning with new employee orientation processes, and used in combination with other implementation strategies that enable active learning (e.g., coaching/consultation, practice opportunities) to advance needed practice changes in clinic milieus (51, 52). For providers and staff in bustling clinics, continuing education credits and the use of small-group training formats that are dynamic and of short duration may incentivize participation and lessen concerns over lost productivity.

While an increasing number of curricular hours in medical education address LGBTQ+ health (53), overall confidence in clinical skills remains low across health professional trainees (54). Notably, exceedingly few curricula in health professions training teach clinical skills required to care for LGBTQ+ patients (55), even though clinical skills-based training is believed to be one of the most effective educational methods influencing clinical care (56). Thus, it is not surprising that our study's direct service providers felt ill-equipped and believed that additional education could help. Given that patient care is currently being impacted, we further recommend focusing education specifically on clinical skills for providers. Alongside other recommendations about improving the quality of care and metrics for quality, we also suggest pairing clinical skills training directly with the skills required to improve markers of health quality.

Finally, participants pointed to the value of the perspectives of LGBTQ+ community members as a key to reducing patient access barriers. Participants critiqued their workplaces for insufficient outreach to LGBTQ+ people, suggesting that such individuals can help determine what is working well and what may be needed to better support services for LGBTQ+ patients. Some participants proposed collecting data directly from LGBTQ+ patients to help plan for potential changes. In this vein, outreach and support for LGBTQ+ people in clinics and communities can be prioritized by encouraging relationship building with community coalitions and organizations focused on LGBTQ+ people, taking part in local LGBTQ+ events, increasing access to information about LGBTQ+ health and healthcare, local advertising, and supporting public policies to reduce health disparities among LGBTQ+ people (4, 20, 57). These activities may be useful for connecting LGBTQ+ patients to services in clinics and resources within communities. Clinics can engage LGBTQ+ patients in other ways as well, including in implementation teams and governance or advisory boards, to guide primary care improvements.

Findings from this baseline analysis informed the development of an online toolkit for implementing LGBTQ+ inclusive guidelines in primary care clinics (20). The toolkit applies an implementation science perspective and provides detailed descriptions of steps clinics can take, along with resources, to promote and evaluate LGBTQ+ inclusiveness and competence at multiple service delivery levels. Our future research will shed further light on how clinics fare in improving care for LGBTQ+ patients through implementation of the guidelines. Other relevant resources include quality improvement initiatives, such as “Transforming Primary Care for LGBT People,” to cultivate the capacity for culturally affirming care for gender and sexual minorities in FQHCs (21, 57, 58).

## Limitations

Generalizability is limited by the purposive sample of five clinics in a single state and the small number of participants. We refrained from recruiting providers/staff directly to minimize burdens on clinic administrators, i.e., compiling employee contact information. Consequently, the recruitment strategy may have led to an underrepresentation of employees harboring negative LGBTQ+ patient sentiment, an overrepresentation of employees concerned about doing so or with vested interests in portraying themselves and the places they work in positively, and fewer participants. Two clinics had services for gender minority patients and personnel knowledgeable about this population. While there is the possibility of social-desirability bias, participants in these and the other clinics nonetheless revealed multiple areas for improving primary care for LGBTQ patients, with findings resonating with those from other recent research (58). All clinics were safety nets serving large and diverse patient populations and subject to high demand for services and federal mandates that substantially influence how services are prioritized and delivered to receive needed funding. The perceptions and experiences of employees in for-profit or private practice settings may be different than those described here. Finally, we applied the R = MC^2^ heuristic during the analysis process after collecting all data. Closer alignment of data collection with the heuristic would likely add to findings on readiness.

## Conclusion

As a cornerstone of our healthcare system, primary care must include essential services for LGBTQ+ patients. This study demonstrates that primary care personnel in safety-net clinics support initiatives to enhance service environments, policies, and practices for LGBTQ+ patients. However, beliefs about treating all patients the same and time/resource constraints may lessen motivation and capacity in busy, understaffed work settings with a diverse clientele. Efforts are needed to overcome structural injustice by removing barriers and building general and innovation-specific capacities for initiatives related to LGBTQ+ patients. Implementation teams, organizational assessments, education, resource allocation, leadership support behaviors, community engagement, and top-down incentives are critical to building such capacities. The R = MC^2^ heuristic may be useful for conceptualizing and enacting strategies to improve readiness and ensure that clinics are ripe for implementing and sustaining policies and practices for decreasing health and healthcare disparities for LGBTQ+ people.

## Data availability statement

The data that support the findings of this study are available from the corresponding author (CW) upon reasonable request.

## Ethics statement

This study involving human subjects was reviewed and approved by the Pacific Institute for Research and Evaluation Institutional Review Board. The participants provided written informed consent to take part in the study.

## Author contributions

CW, RS, KE, and MK conceptualized the study on which this manuscript is based. CW, RS, and MK collected data. SD organized the database. CW analyzed the qualitative data and wrote the first draft of the manuscript. MS analyzed the quantitative data. MS, KE, and MK wrote sections. CW, MS, KE, RS, SD, and MK contributed to mixed-method data triangulation and interpretation of findings. All authors revised the manuscript and read and approved the submitted version.

## Funding

This work was supported by a grant from the U.S. National Institute of Minority Health and Health Disparities (R21MD011648). The funding source had no role in the design of this study, its execution, analyses, interpretation of the data, and decision to submit results.

## Conflict of interest

The authors declare that the research was conducted in the absence of any commercial or financial relationships that could be construed as a potential conflict of interest.

## Publisher's note

All claims expressed in this article are solely those of the authors and do not necessarily represent those of their affiliated organizations, or those of the publisher, the editors and the reviewers. Any product that may be evaluated in this article, or claim that may be made by its manufacturer, is not guaranteed or endorsed by the publisher.
